# Diffuse Reflectance Infrared Fourier Transform Spectroscopy for the Determination of Asbestos Species in Bulk Building Materials

**DOI:** 10.3390/ma7010457

**Published:** 2014-01-16

**Authors:** Grazia Accardo, Raffaeke Cioffi, Francesco Colangelo, Raffaele d’Angelo, Luca De Stefano, Fderica Paglietti

**Affiliations:** 1 Centro Direzionale Isola C4, Università di Napoli Parthenope, 80132 Napoli, Italy; E-Mails: r.cioffi@uniparthenope.it (R.C.); colangelo@uniparthenope.it (F.C.); 2 INAIL-Direzione Regionale della Campania, Via Nuova Poggioreale, 80132 Napoli, Italy; E-Mail: r.dangelo@inail.it; 3 CNR-IMM, Istituto per la Microelettronica e Microsistemi, Via Pietro Castellino 111, 80131 Napoli, Italy; E-Mail: luca.destefano@na.imm.cnr.it; 4 INAIL-DIPIA Dipartimento Installazioni di Produzione ed Insediamenti Antropici, Via Urbana 167, 00184 Roma, Italy; E-Mail: f.paglietti@inail.it

**Keywords:** asbestos, analytical chemistry, FTIR spectroscopy

## Abstract

Diffuse reflectance infrared Fourier transform (DRIFT) spectroscopy is a well-known technique for thin film characterization. Since all asbestos species exhibit intense adsorptions peaks in the 4000–400 cm^−1^ region of the infrared spectrum, a quantitative analysis of asbestos in bulk samples by DRIFT is possible. In this work, different quantitative analytical procedures have been used to quantify chrysotile content in bulk materials produced by building requalification: partial least squares (PLS) chemometrics, the Linear Calibration Curve Method (LCM) and the Method of Additions (MoA). Each method has its own pros and cons, but all give affordable results for material characterization: the amount of asbestos (around 10%, weight by weight) can be determined with precision and accuracy (errors less than 0.1).

## 1. Introduction

Italy is a country covering a surface area of 301,340 km^2^, with about 60,626,442 inhabitants and a population density of 201 inhab./km^2^. It is thus a very densely populated country, with much of its territory urbanized and requiring careful protection of valuable cultural or natural assets.

This country has been one of the biggest producers of raw asbestos, by mining, and asbestos containing material (ACM), especially for building applications. Asbestos is a common name for classifying a family of silicate minerals, which are sub-divided in amphibole and layer-silicate asbestos on the basis of their structures and chemical compositions [1,2]. Only five species of asbestos are regulated by Italian law, due to their established hazard; namely, anthophyllite, grunerite, crocidolite and tremolite varieties belong to the amphibole group, while chrysotile belongs to the serpentine group. Even if crocidolite is by far the most hazardous kind of asbestos, chrysotile accounts for over 90% of the world’s consumption of this material, so we focused our investigation on this world-wide diffused silicate [Mg_3_Si_2_O_5_(OH)_4_].

Italy was the first European country banning asbestos in 1992: the extraction, import, export, marketing and fabrication of asbestos-based products were forbidden [3,4]. Among the European Community, Italy has one of the most restrictive laws about asbestos.

Legal prescriptions, published over the last 20 years, covered the main items in occupational fields, such as personal and work environment exposure, threshold limits values (sampling, analysis, *etc.*), technical standards for remediation, mapping, classification of asbestos containing waste (ACW), landfill management, inertization and re-use methods, and so on. Due to its chemical composition, inertized ACW can be used as a filler in building materials [5]; concrete and mortar mixtures are good matrices for waste immobilization [6–10]. Furthermore, a lot of studies dealt with the pathogenic mechanisms by which asbestos act [11].

From the analytical point of view, the Italian Ministerial Decree issued on 6 September 1994, indicates some analytical techniques and methods for qualitative and quantitative ACM characterization: X-ray diffraction (XRD), Fourier transform infrared spectroscopy (FTIR) and scanning electron microscopy (SEM), but gives a specific description of the analytical procedures only in case of XRD and SEM. XRD can detect the content of asbestos with a detection limit of 10% weight by weight (*w*/*w*); SEM is able to reveal asbestos traces (less than 0.1% *w*/*w*). The FTIR method is still not officially defined. It is well known that different types of asbestos can be identified and quantified by means of FTIR spectroscopy: strong and specific sharp peaks can be found in the absorption spectra in the 4000–400 cm^−1^ wavenumber region [12,13]. Several studies have demonstrated that FTIR is suitable for the quantification of micrograms of different kinds of asbestos in host matrices, and the FTIR limit of detection could be even less than 1% *w*/*w* in the case of bulk samples [14,15]. For these reasons, although samples require careful pre-treatment before analysis, FTIR could be an alternative approach with respect to other used techniques [16–19]. In our recent paper, we have compared different analytical FTIR procedures for a low content of asbestos in bulky materials [20], also focusing our attention on sample preparation: classic FTIR measurements are done on powders dispersed in potassium bromide (KBr), which is transparent to infrared radiation. The way powders are obtained by massive ACM is simple milling (both by hands or automatic), but it is not a trivial process: the powder size should be less than one micron on average, and the milling step should not damage the fiber structure; otherwise, the result is false [21]. Some FTIR related techniques, like diffuse reflectance infrared Fourier transformed (DRIFT) spectroscopy, can be applied to non-transparent media and could avoid sample milling, thus being very attractive for rapid and *in situ* asbestos analysis; on the other hand, since DRIFT is a surface-limited analytical technique, the detection limit is larger than the FTIR classical one. In this paper, we compared different quantitative procedures applied to the DRIFT spectra of ACM and demonstrated that this technique is well suited for quantifying asbestos content in bulk material with high precision and accuracy, at least in ACM coming from building remediation, containing between 10% and 20% *w*/*w* of asbestos.

## 2. Theory

DRIFT is a surface localized FTIR spectroscopy, since it can provide both chemical and structural information for all types of solid surfaces. When infrared radiation reaches a sample surface, one or several processes can occur: light can be adsorbed, reflected from the surface, or it can penetrate the sample before being scattered. If scattering centers, which are fibers in the case of ACM, are randomly oriented, the phenomenon is isotropic and generates a diffuse reflectance [22]. The scattered light is then collected and relayed to the IR detector, where the absorption by chemical groups is revealed. DRIFT spectrometry has many advantages with respect to the conventional transmission (or reflection) FTIR method: DRIFT is a fast and non-destructive technique, since the sample can be directly analyzed, both as it is or in its powdered form; moreover, DRIFT is better suited to the analysis of strongly absorbing materials, whose main characteristic are very low signal and sloping baselines when analyzed in transmission. Since the optical phenomena that generate DRIFT signals are different from those involved in transmittance spectrometry, the spectra obtained by these methods cannot be considered equivalent. It is therefore mandatory to verify that DRIFT, which is a powerful experimental technique, can be usefully used as an analytical quantitative method in measuring the amount of asbestos contained in bulk materials.

All type of asbestos minerals exhibit strong absorption in the 1200–900 cm^−1^ band, due to Si–O stretching vibration and in 600–900 cm^−1^, due to vibration of the silicate chain, to metal-oxygen stretching and Si–O bending vibration [23,24]. O–H stretching vibrations give distinct peaks in the region 3600–3700 cm^−1^ [25]. In addition, O–H bending frequencies lie in the 950–600 cm^−1^ region [26]. The linear absorbance (in the following, always referred to as the peak height), or the area, of strong absorption peaks, called, for this reason, “analytical” peaks, can be used to quantify the weight of asbestos, expressed in micrograms or in concentration weight by weight (*w*/*w*). The analytical peaks for different types of asbestos are reported in Table 1 [27–29]. Chrysotile shows double O–H peaks stronger at about 3685 cm^−1^ and weaker at 3655 cm^−1^ [26]. A broad O–H bending band is present at 605 cm^−1^, Si–O stretching vibration at 1069, 1033 and 959 cm^−1^, and evident bands at about 606, 434 and 300 cm^−1^ can be assigned to Mg–OH bending frequency [20,25].

In this work, we have applied and compared three different analytical procedures (the Method of Addition, partial least squares and the Linear Calibration Curve Method) for the quantitative determination of a few micrograms of asbestos in bulk materials.

### 2.1. Method of Additions

Quantitative analyses using calibration curves are based upon measuring a property of a sample that changes with the analyte concentration [20]: in the present case, the calibration curves are obtained by plotting the height of the absorption peak, *h_M_*, as a function of the concentration, *C_x_*; *i.e.*, *h_M_ = A + B C_x_*. The Method of Addition (MoA) is a classic internal standard method developed for the quantitative determination of an analyte in a complex matrix and, therefore, is less dependent on the matrix composition and complexity [15]. The calibration curve is calculated by evaluating *h_M_* for a series of mixtures obtained by multiple additions of known quantities of standard asbestos to the original matrix. The standard asbestos was provided by the USA National Institute for Standards and Technology (SRM 1866b, nominal concentration by weight greater than 90%, CAS Number: 12001-29-5). The unknown weight concentration of asbestos in the sample is given by the intersection between the calibration line and the *x*-axis, *i.e.*:

$${\bar{C}}_{x}=\frac{A}{B}$$ (1)

The relative error is, in this case:

$$\varepsilon_{r}=\frac{\delta A}{\left|A\right|}+\frac{\delta B}{\left|B\right|}$$ (2)

where δ*A* and *B* are relative errors on *A* and *B*, respectively.

Once the calibration curve has been calculated, the concentration of an unknown sample of analyte is evaluated by measuring the response of the unknown asbestos sample under the same conditions as used for the standard. From the mathematical point of view, MoA is a regression line formula, and for this reason, its intrinsic precision could be limited with respect to other analytical procedures [30].

### 2.2. Partial Least Square

The Partial Least Squares (PLS) Method is currently used to solve both descriptive and predictive problems in experimental life sciences, especially in chemistry [31–33]. In a few words, PLS connects two generic data matrices, *X* and *Y*, by a linear multivariate model, allowing the analysis of data with many, noisy, collinear, and, even, incomplete variables in both *X* and *Y*. PLS precision of model parameters estimation improves by increasing the number of relevant variables and observations [34–38]. This statistical approach can be used to predict the properties of interest, based on the measurement of chemical system observables, such as an infrared spectrum, but it also holds for Raman or nuclear magnetic resonance spectra. A detailed description of how PLS works is out of the scope of this work, so we can only underline that the goal of PLS regression is to predict values of *Y* (namely, concentrations of asbestos in different samples) from known values of *X* (namely, the analytical peak heights of samples and reference standards), but there are, in the literature, a lot of PLS applications for chemometrics, *i.e*., the quantification of chemical quantities through statistical methods [30,35–44].

### 2.3. Linear Calibration Curve Method

LCM is commonly used in quantitative chemistry, and it has been adopted successfully for the determination of asbestos in bulk materials by means of XRD [20]. This analytical procedure can be also used in DRIFT quantitative spectrometry; also in this case, the curves are obtained by plotting the height of the absorption peak, *h_M_*, as a function of the concentration, C*_x_* (*w*/*w*), of the analyte, but sample mixtures contain known quantities of a given standard of asbestos together with one or two materials that mimic a matrix (in our case, a commercial concrete; see also the Experimental section) [16,45]. Since the standard is not added, but it is used as a sample itself, LCM is an external standard method, and it can work well only if the standard is really the same material as that contained in the ACM.

The calibration curve can always be approximated to a straight line, at least for very small ranges of concentration, the equation of which is, again: *h_M_* = *B C_x_* + *A*. It is therefore possible to find the best line that interpolates the experimental points and that determines the line parameters, *A* and *B*, and also the relative errors, δ*A* and δ*B*. The unknown concentration of asbestos in the analyzed sample is determined by using the following formula:

$${\bar{C}}_{x}=\frac{h_{M}-A}{B}$$ (3)

Relative and absolute errors on this quantity, ε_r_ and ε_a_, respectively, are calculated by the usual formulas of error propagation:

$$\varepsilon_{r}=\frac{\delta h_{M}+\delta A}{\left|h_{M}-A\right|}+\frac{\delta B}{\left|B\right|}$$ (4)

$$\varepsilon_{r}=\varepsilon_{a}{\bar{C}}_{x}$$ (5)

where the error on *h_M_*, δ*h_M_*, is the statistical indetermination of several independent measurements on the same sample. Even if LCM is largely adopted in spectroscopy, quantitative results from the LCM procedure can be strongly affected by the interference of the matrix in which the asbestos is embedded. As happens with most spectrometric techniques, some peaks of the analyte can be partially or fully masked by other substances’ peaks, preventing correct quantitative measurements [46].

### 3. Results and Discussion

**Figure 1.**
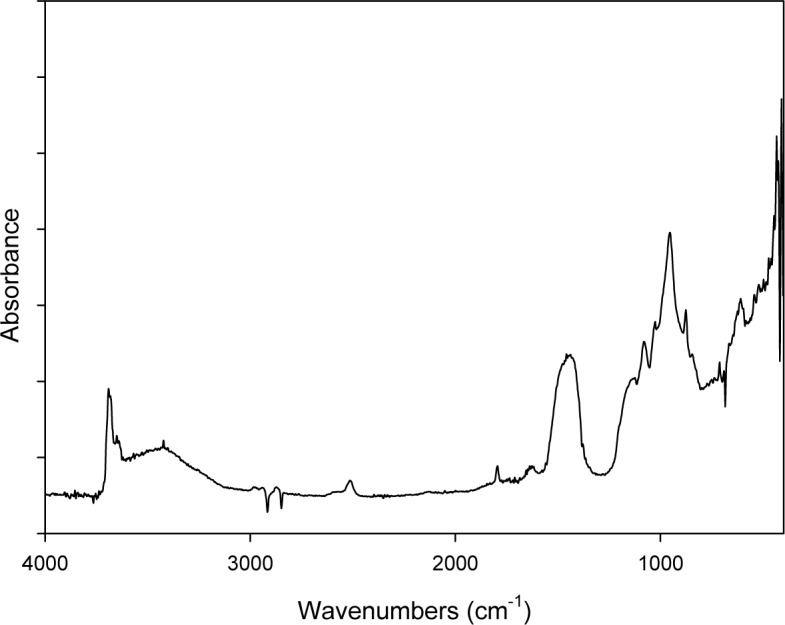
Diffuse reflectance infrared Fourier transform (DRIFT) spectrum of the asbestos containing material (ACM) sample.

Figure 1 shows the DRIFT spectrum of ACM powders: the characteristic doublet absorption peaks at 3688 cm^−1^ and 3645 cm^−1^ of chrysotile are evidenced well. Other peaks are ascribed to the C–O (2924–2854 cm^−1^; 2400–2200 cm^−1^ and 1720 cm^−1^), Si–O–Si (1090–1000 cm^−1^) and S–O (1200–650 cm^−1^) chemical groups present in the compound that constitutes the cement matrix in which the asbestos is embedded (see also the ACM description in the Experimental section).

**Figure 2.**
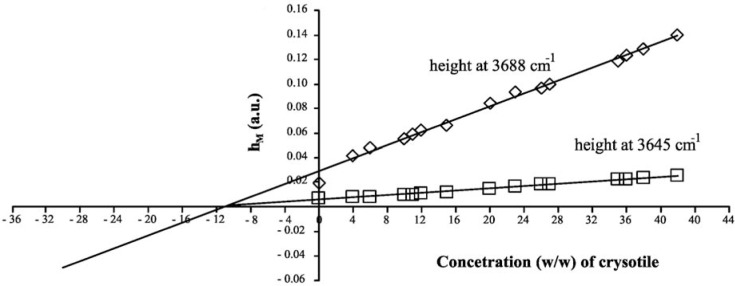
The Method of Additions (MoA) linear curves of ACM.

The MoA linear curves are shown in Figure 2; the relevant parameters are reported in Table 2. The *x*-intercept of the MoA plot corresponds to the unknown amount of analyte that should be present in the sample. Since chrysotile shows two characteristic absorption peaks that can be used as analytical references, two MoA curves can be elaborated, one for each peak: of course, both the results should be the same if there are no systematic errors in the quantitative procedure. By elaborating data referring to the 3688 cm^−1^ peak, an asbestos concentration of 11.23% ± 0.06% is estimated; in the case of a peak at 3645 cm^−1^, the concentration is calculated as 11.20% ± 0.05%. This result demonstrates that the DRIFT technique could be simple, fast and also very accurate and precise.

In Table 3, the composition of the mixtures used for the calibration and validation steps required by the PLS-based chemometric procedure is reported (see the Experimental section for a brief explanation of how the TQ Analyst software works).

**Figure 3.**
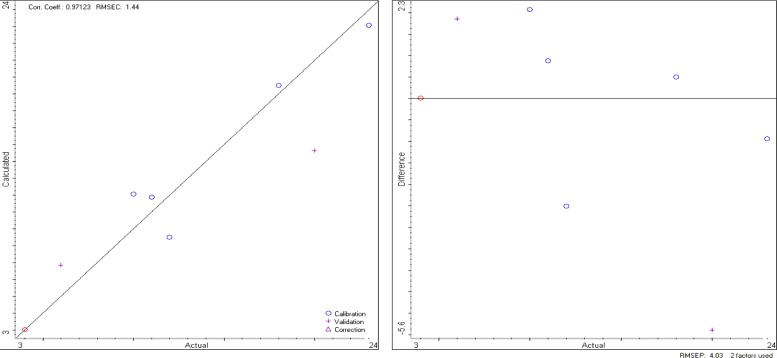
TQ Analyst software calibration and validation curves.

The TQ Analyst software elaborates data from the DRIFT spectra and, as a result, produces a calibration curve and a validation curve, as reported in Figure 3.

If we apply the calibration model to the unknown sample, previously analyzed by MoA, we obtain the results reported in Table 4, which are very close to that of MoA, even if the errors are slightly larger.

**Figure 4.**
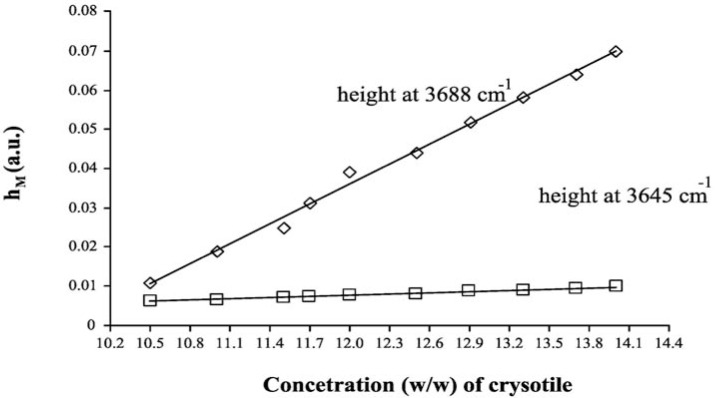
Linear Calibration Curve Method (LCM) linear curves for ACM.

The LCM curves, calculated for both analytical peaks of chrysotile, are shown in Figure 4, while the curve parameters (slope, intercept and their errors) are summarized in Table 5. One basic assumption for the analytical method proposed is that the relationship between *h_M_* and *Cx* should be linear. From the plot reported in Figure 4, it was experimentally verified that in the concentration range explored, the dependence of *hM* on *Cx* is actually linear: the linear regression coefficient of both curves reported in Figure 4 is greater than 0.99. The linearity assumption is not true in general, and the relationship should be verified case by case. It is better to prepare the calibration curves in those intervals of concentration where the linearity is experimentally measured.

As in the case of MoA, we can quantify the asbestos content in the ACM sample by using the curve parameters of both peaks at 3688 cm^−1^ and 3645 cm^−1^, by using the formulas reported in the Theory section, we obtained 11.13% ± 0.02% and 11.30% ± 0.07%, respectively. These numbers show that also, in the case of LCM, the DRIFT spectroscopy is precise and accurate.

As the first conclusion, we can state that from the quantification point of view, the three analytical procedures are equivalent, since in all the experiments that were realized, it was found that the chrysotile content was the same within the experimental errors. For all three methods, the sample preparation and data acquisition procedure are important in order to maximize accuracy and precision. While MoA and LCM require peak height calculation, which means the individuation of peaks and baseline correction, PLS is completely automated, *i.e*., the software analyzes the spectrum and makes all the calculations. Nevertheless, we should emphasize that the chemometric method is simple, fast and equivalent to MoA and LCM in terms of the results, only when the correlation matrix has a few components; but in the case of many components, the chemometric method implies too much complex calculations, requiring a long time.

The need of a critical comparison between different FTIR methodologies, highlighting the pros and cons of each one, arises form a lack of technical prescription in Italian regulation: while X-ray diffraction and optical, as well as electronic, microscopy are well described, FTIR is allowed, but not standardized. We believe that in the near future, there will be a strong need of fast, accurate and precise quantitative methods for monitoring ACM and also asbestos containing wastes or contaminated soils; in Italy there are 10 Superfund, *i.e*., especially contaminated locations, and more than 34,000 sites mapped and inserted in a specific database, where asbestos is still in place and needs some kind of remediation action.

In this framework, DRIFT spectroscopy represents a rapid-screening method for the quantification and classification of materials. The procedures tested in this work can be successfully applied to different bulk asbestos materials for the qualitative and quantitative determination of all types of asbestos.

## 4. Experimental Section

Asbestos containing materials (ACM), were supplied by “Ambiente s.r.l.” in the framework of a local research project “Progetto Rifiuti” (INAIL, Regional Direction for Campania) and came from the remediation of industrial building roofs. The ACM was classic cement reinforced by asbestos fibers; this kind of material has been deeply characterized by standard chemical and structural techniques (in our case, X-ray diffraction using a Philips PW3020 X’Pert Diffractometer under the following operating conditions: Bragg–Brentano configuration, Θ–2Θ; tension, 40 kV; current, 40 mA; anode, copper; scanning, step; step size, 0.01°; time per step, 1 s.; data not shown here); and the compositional information agrees with that supplied by the technical sheet of the manufacturer: chrysotile, calcium carbonate, calcium sulfate and alumina. In such materials, the asbestos content was always between 10% and 15%, weight by weight. ACM handling requires special health security procedures: ACMs were received sealed in double polyethylene bags; each bag was opened inside a laminar flow hood to prevent any fiber dispersion into the laboratory, according to the Italian Environment Ministry Decree 6 September 1994, and related acts. Researchers, during laboratory activity, wore protective disposable full-body overalls and the prescribed facial masks. ACM was gently dry-crushed in an agate mortar and then finely ground in a ring mill (FRITSCH, model Pulverisette 9, rotational speed 750/1000 rpm) enclosed in a vial. In all the above operations, the air was monitored by filtration through cellulose filters and fibers counted using phase contrast optical microscopy (PCOM). Fiber concentration, in all analyses, never exceeded the threshold limit (100 fibers/L).

### Quantitative Analysis of Asbestos

An FTIR spectrometer Nicolet 6700 (Thermo Nicolet Corp., Madison, WI, USA) equipped with a diffuse reflectance accessory (DRIFT, Thermo Nicolet Corp., Madison, WI, USA) was used to obtain the FTIR spectra of the samples. Spectra were collected in the range 4000–400 cm^−1^ by 32 scans and at resolution of 4 cm^−1^. All spectra were analyzed by the software of the OMNIC operating system (Version 7.0 Thermo Nicolet, Thermo Nicolet Corp., Madison, WI, USA) and normalized against an air background. After every measurement, a new reference air background spectrum was taken.

#### (1) Regression with Method of Additions

Mixtures of ACM and known quantities of standard chrysotile (NIST standard SRM 1866b) were prepared by multiple additions in the range of 4%–42% weight by weight. Samples were mixed and homogenized in an agate mortar for a few minutes. The heights of the chrysotile characteristic peaks at 3688 cm^−1^ and 3645 cm^−1^ were registered for each addition, and the data were plotted for linear regression. An unknown asbestos concentration is obtained by the intersection of the best-fit line and the *x*-axis.

#### (2) Regression with PLS

The software, TQ Analyst, was used for PLS. This statistical analysis requires two elaboration steps: calibration and validation. In the calibration procedure, the software searches for a relation between the dependent variable, *Y* (peak height), and the independent variable, *X* (asbestos concentration), which can be generically written as: *Y* = *f*(*X*1, *X*2, *X*3, …, *Xp*). In practice, an algorithm, based on partial least squares equations, calculates the regression coefficients of the equation: *Y* = b0 + b1*X*1 + b2*X*2 + …. bp*Xp*; which define the mathematical model of the system under investigation. The second step is a so-called “leave-one-out” cross-validation procedure that is used to verify the calibration model: the FTIR spectra of the standard sample, containing a known amount of asbestos, are elaborated by the TQ software and the results used to evaluate the goodness of the mathematical model. If the model produces positive results during the validation, it can be used to obtain the quantification of unknown samples. DRIFT spectra have been smoothed, and the wave number range has been set around the analytical peaks of chrysotile, in order to minimize the number of calculations in partial least squares. Smoothing is also mandatory, since ground noise can confuse the algorithm.

#### (3) Linear Calibration Curve Method

The Linear Calibration Curve Method (LCM) is commonly used in quantitative chemistry, and it has been adopted successfully for the determination of asbestos in bulk materials by means of XRD [47,48]. This analytical procedure can be also used in FTIR quantitative spectrometry: in this case, the calibration curves are obtained by plotting the height of the absorption peak as a function of asbestos concentration in different mixtures containing known quantities of standard asbestos (NIST SRM 1866b). Different aliquots of standard chrysotile in the range of 10%–14% weight by weight has been mixed with asbestos-free commercial concrete. Samples were mixed and homogenized in an agate mortar for a few minutes. The heights of the chrysotile characteristic peaks at 3688 cm^−1^ and 3645 cm^−1^ were registered for each mixture and the data plotted against the chrysotile concentration for linear interpolation. The parameters, and their errors, of the best-fit linear curve can be used for the estimation of unknown samples.

## 5. Conclusions

In conclusion, the analytical procedures investigated for the quantitative determination of asbestos in bulk materials based on DRIFT spectroscopy are precise and accurate in the explored range of asbestos concentration, even if DRIFT does not allow for a very low (about 1% *w*/*w*) limit of detection. The data acquisition methodology and data analysis require attention for guaranteeing the quality of the outputs. DRIFT spectroscopy can be thus considered very appealing as an analytical technique for bulk determination. Considering the big number of asbestos remediation activities still at work and already previewed and programmed by the Italian Superfund Program (M.D. 468/01) and the Asbestos Mapping Act (M.D. 101/03), as well as the Italian National Asbestos Program for the next five years, which has been approved in May 2013, by the Italian Parliament, it is possible to appreciate the importance of FTIR analytical methodologies and their potentiality.

## Figures and Tables

**Table 1.** Absorption peaks for different types of asbestos.

Type of asbestos	Analytical band (1) (cm^−1^)	Analytical band (2) (cm^−1^)	Analytical band (3) (cm^−1^)	Analytical band (4) (cm^−1^)
Chrysotile	3697–3686–3650–3640	1078–1020–960	654–615–605–550–481–450–440–432	400–305
Amosite	3656–3640–3618	1128–1082–996–981	775–750–703–638–528–498–481–440	385
Crocidolite	3636–3620–3610	1143–1110–939–897	778–775–770–725–694–668–636–630–540–504–495–450	320

**Table 2.** MoA curve parameters.

Analytical band (cm^−1^)	*h*	*B* ± δ*_B_*	*A* ± δ*_A_*	*R*^2^
3688	0.021	0.00260 ± 6 × 10^−5^	0.0292 + 0.0015	0.9928
3645	0.006	4.76 × 10^−4^ ± 2 × 10^−5^	0.0056 + 0.0002	0.9919

**Table 3.** Mixtures used in the partial least squares (PLS) estimation.

Index	Spectrum title	Usage	% Chrysotile	% ACM
1	STANDARD1	CALIBRATION	4.0	96.00
2	STANDARD2	VALIDATION	6.00	94.00
3	STANDARD3	CALIBRATION	10.00	90.00
4	STANDARD4	CALIBRATION	11.00	89.00
5	STANDARD5	CALIBRATION	12.00	88.00
6	STANDARD6	CALIBRATION	18.00	82.00
7	STANDARD7	VALIDATION	20.00	80.00
8	STANDARD8	CALIBRATION	23.00	77.00
9	STANDARD9	CALIBRATION	26.00	74.00
10	STANDARD10	CALIBRATION	27.00	73.00
11	STANDARD11	CALIBRATION	35.00	65.00
12	STANDARD12	CALIBRATION	36.00	64.00
13	STANDARD13	CALIBRATION	38.00	62.00
14	STANDARD14	CALIBRATION	42.00	58.00

**Table 4.** PLS results.

Index	Component	Concentration	Unit	Uncertainty
1	Chrysotile	11.06	%	9.461
2	ACM	88.94	%	9.461

**Table 5.** LCM curve parameters.

Analytical band (cm^−1^)	*h*	*B* ± δ*_B_*	*A* ± δ*_A_*	*R*^2^
3688	0.021	0.0169 ± 0.0004	−0.167 ± 0.005	0.9945
3645	0.0069	0.0010 ± 0.0002	−0.0438 ± 0.0002	0.9962
